# Effects of Short-Term Phosphate Loading on Aerobic Capacity under Acute Hypoxia in Cyclists: A Randomized, Placebo-Controlled, Crossover Study

**DOI:** 10.3390/nu14020236

**Published:** 2022-01-06

**Authors:** Kamila Płoszczyca, Małgorzata Chalimoniuk, Iwona Przybylska, Miłosz Czuba

**Affiliations:** 1 Department of Kinesiology, Institute of Sport, 01-982 Warsaw, Poland; Milosz.czuba@awf.edu.pl; 2 Department of Physiotherapy, Faculty of Physical Education and Health in Biala Podlaska, Jozef Pilsudski University of Physical Education in Warsaw, 21-500 Biala Podlaska, Poland; mchalim@yahoo.com (M.C.); iwona.przybylska@awf.edu.pl (I.P.); 3 Faculty of Rehabilitation, Jozef Pilsudski University of Physical Education in Warsaw, 00-968 Warsaw, Poland

**Keywords:** phosphate loading, ergogenic aids, hypoxia, altitude, aerobic capacity, 2,3-diphosphoglycerate, hemoglobin oxygen affinity, buffering capacity, hypoxia-inducible factor 1 alpha, athletes

## Abstract

The aim of this study was to evaluate the effects of sodium phosphate (SP) supplementation on aerobic capacity in hypoxia. Twenty-four trained male cyclists received SP (50 mg·kg^−1^ of FFM/day) or placebo for six days in a randomized, crossover study, with a three-week washout period between supplementation phases. Before and after each supplementation phase, the subjects performed an incremental exercise test to exhaustion in hypoxia (FiO_2_ = 16%). Additionally, the levels of 2,3-diphosphoglycerate (2,3-DPG), hypoxia-inducible factor 1 alpha (HIF-1α), inorganic phosphate (Pi), calcium (Ca), parathyroid hormone (PTH) and acid-base balance were determined. The results showed that phosphate loading significantly increased the Pi level by 9.0%, whereas 2,3-DPG levels, hemoglobin oxygen affinity, buffering capacity and myocardial efficiency remained unchanged. The aerobic capacity in hypoxia was not improved following SP. Additionally, our data revealed high inter-individual variability in response to SP. Therefore, the participants were grouped as Responders and Non-Responders. In the Responders, a significant increase in aerobic performance in the range of 3–5% was observed. In conclusion, SP supplementation is not an ergogenic aid for aerobic capacity in hypoxia. However, in certain individuals, some benefits can be expected, but mainly in athletes with less training-induced central and/or peripheral adaptation.

## 1. Introduction

Acute exposure to hypoxia leads to a reduction in maximal oxygen uptake (VO_2max_) and endurance exercise performance in athletes [1,2,3,4]. The decrease in VO_2max_ in hypoxia is a consequence of a drop in blood oxygen partial pressure and blood oxygen saturation (SpO_2_), which results in a reduction in oxygen supply to tissues [3,5]. It was found that each 1% decrement in SaO_2_ below the 95% level causes a ∼1–2% reduction in VO_2max_ [6]. At an altitude of ~2000 m, a decrease in endurance performance by 8–10% may be expected [4,7,8,9]. Since the magnitude of the decrease in aerobic performance shows considerable individual variation [10], in more vulnerable athletes, a decrease may also occur at altitudes even below 1000 m [11,12]. Despite many years of research on human adaptation to altitude, the current research issue is the search for methods of limiting the decline in exercise capacity due to acute hypoxic exposure. In sports practice, before competitions at altitude, training camps are organized at terrestrial altitude or under normobaric hypoxia [13,14] in order to activate adaptive mechanisms in athletes [15]. However, adaptation to altitude requires a sufficiently long duration. It is recommended that the acclimation period should be at least 14 days before competition at ~2000 m [14,16].

Dietary supplements potentially support the improvement of exercise capacity in hypoxic conditions and optimize acclimatization to altitude [17,18]. Phosphate salts, due to their mechanism of action, seem to be a promising and legal nutritional ergogenic aid for athletes exercising in hypoxia. The potential benefits of phosphate loading include enhanced 2,3-diphosphoglycerate (2,3-DPG) concentrations in erythrocytes, resulting in an improvement in oxygen unloading in muscles by reducing hemoglobin oxygen affinity (Hb-O_2_ affinity), an increase in myocardial efficiency, an improvement in buffering capacity and acceleration of the synthesis and restoration of adenosine triphosphate (ATP) [19].

Studies conducted under normoxic conditions have found that phosphate salt supplementation leads to a 3–12% increase in VO_2max_ and an improvement in aerobic exercise performance [20,21,22,23,24,25]. However, not all research supports these beneficial phosphate loading effects [26,27,28,29] and recommendations for the use of phosphates by athletes remain ambiguous [19,30,31]. Research into the effects of phosphate salts under hypoxic conditions is scarce. It is recognized that hypoxic exposure leads to an increase in 2,3-DPG level and a decrease in Hb-O_2_ affinity [32,33,34]. Early studies showed that these adaptations could be enhanced by phosphate loading. Moore and Brewer [35] found that after 24 h at an altitude of 4300 m, the level of 2,3-DPG was higher in the phosphate-supplemented group than in the placebo group. Jain et al. [36] reported that during a stay at an altitude of 3500 m, short-term (4 days) supplementation with sodium phosphate led to an 18% increase in blood levels of 2,3-DPG in male sea-level residents, which was beneficial for short-term high altitude adaptation. Furthermore, Moore et al. [37] noted that phosphate loading led to an increase in 2,3-DPG levels by 15% before ascent to altitude (3500 m) and maintenance of high 2,3-DPG levels during the initial period of altitude exposure. The observed changes were accompanied by an improvement in central nervous system function [35,36,37]. However, the studies failed to analyze the effect of phosphate salts ingestion on the exercise performance of the participants. Furthermore, these experiments were conducted at high altitude, which may affect the adaptive response differently than at moderate altitude. Over the decades, the analysis of the ergogenic effect of sodium phosphate in hypoxia was not continued. In our latest study [38], we observed that 6 days of sodium phosphate supplementation promoted improvements in the efficiency of the cardiorespiratory system in cyclists during low- and moderate-intensity exercise in a hypoxic environment (FiO_2_ = 16%, ~2500 m). Based on the same methodology, in the present study we analyze the effects of short-term (6 days) sodium phosphate (SP) supplementation in trained cyclists on (1) aerobic capacity under normobaric hypoxia (FiO_2_ = 16%, ~2500 m), (2) the level of 2,3-DPG and hemoglobin oxygen affinity, (3) the buffering capacity, (4) the phosphate-calcium balance. We hypothesize that SP supplementation by increasing the level of 2,3-DPG in erythrocytes will reduce the Hb-O_2_ affinity, consequently leading to the improvement of aerobic capacity in hypoxia. Further, short-term phosphate loading will not disturb calcium-phosphate balance.

## 2. Materials and Methods

### 2.1. Study Participants

Twenty-four trained male cyclists (aged 34.8 ± 4.7 years; body height 181.9 ± 6.7 cm; body mass 73.9 ± 7.2 kg; fat content (%) 13.7 ± 3.4%; fat-free mass (FFM) 63.8 ± 6.6 kg) participated in this study. The basic inclusion criteria were: a minimum of six years of training experience and at least a six-month washout period from previous altitude training and sodium phosphate supplementation. All the participants had recently undergone medical examinations, without any contraindications that would exclude them from the study. The participants provided their written voluntary informed consent before participation. Additionally, the participants declared that for at least one month before testing, they did not take either medications or dietary supplements. Furthermore, before the experiment, blood electrolyte levels were analyzed in the participants under fasting conditions and they were within the reference range in all athletes. The study participants were randomly divided into two equal research groups, G1 and G2, using a computer-generated randomized list [39].

After the first series of tests, one of the participants withdrew from further participation in the experiment due to illness. Additionally, one participant reported gastrointestinal (GI) disturbances (stomach pain) after three days of sodium phosphate supplementation. For this reason, supplementation was discontinued in this athlete and the obtained data were not taken into account in the analysis. Ultimately, twenty-two subjects were included in the statistical analysis. Statistical power analysis (GPower 3.1 software) [40] showed that with such a sample size (*n* = 22), while maintaining an acceptable power (1-β = 0.80) and α = 0.05, the test will detect an effect size (ES) = 0.31 (small).

The research project was conducted according to the Declaration of Helsinki and was approved (no. 4/2018, approval date: 15 November 2018) by the Ethics Committee for Scientific Research at the Jerzy Kukuczka Academy of Physical Education in Katowice, Poland.

### 2.2. Study Design

**Figure 1:**
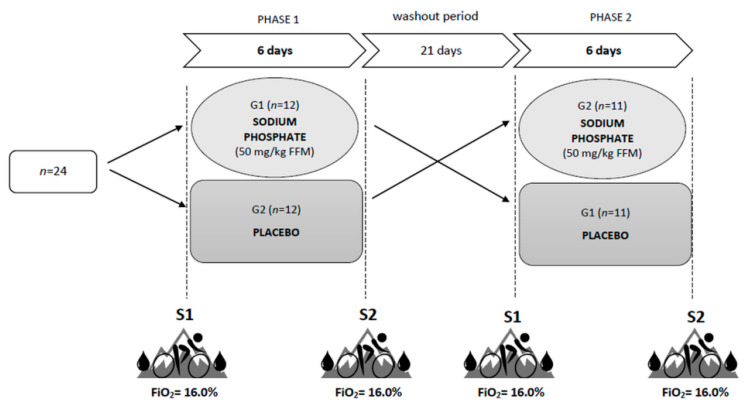
Study design. S1 and S2—two test series, before and after each supplementation phase.

The study was carried out in a crossover design using a placebo procedure (Figure 1). The experiment included two six-day supplementation phases with a twenty-one-day washout period (for details, see the “Supplementation with sodium phosphate” section). Before and after each supplementation phase, two test series (S1 and S2) were carried out. All the series were based on the same methodology. The time of day and the order of the participants were maintained to ensure similar conditions for the measurements. Each test series began by obtaining venous blood (10 mL) from an antecubital vein under fasting conditions to determine the resting levels of the following variables: hemoglobin concentration ((Hb)), hematocrit (Hct), red blood cell count (RBC), reticulocytes (Ret), serum levels of inorganic phosphate (Pi) and calcium (Ca), and parathyroid hormone (PTH). Blood tests were performed by a local laboratory using the Sysmex XN 2000 hematology analyzer (Sysmex, Kobe, Japan), and hydrodynamically focused impedance (RBC, Hct), fluorescence flow cytometry (Ret) and cyanide-free SLS hemoglobin methods ([Hb]). The levels of Pi and Ca were determined by the spectrophotometric method (Cobas 8000 c702 Chemistry System, Roche Diagnostics, Mannheim, Germany), while the electrochemiluminescence (ECL) technology for immunoassay analysis was used to measure the PTH levels (Cobas 6000 e601 immunology analyzer, Roche Diagnostics, Mannheim, Germany). After blood collection, body height, body mass, and body composition were measured using the DXA (dual-energy X-ray absorptiometry) method (GE Lunar Prodigy, GE Healthcare, Madison, WI, USA).

Two hours after a light mixed meal (5 kcal/kg body weight, 50% CHO, 30% fat, 20% protein) and after 15 min of passive exposure to normobaric hypoxia (FiO_2_ = 16%; ~2500 m), venous blood was obtained to determine the resting levels of 2,3-DPG and hypoxia-inducible factor 1 alpha (HIF-1α). Next, the subjects performed an incremental exercise test under normobaric hypoxia conditions (FiO_2_ = 16%; ~2500 m) to determine the values of VO_2max_ and lactate threshold (LT). The incremental test was performed using the Excalibur Sport cycle ergometer (Lode, Groningen, The Netherlands) and was carried out in a normobaric hypoxic chamber (AirZone 25, Air Sport, Warsaw, Poland). During all the test series, the atmospheric conditions, such as temperature (19 °C), humidity (50%), the concentration of carbon dioxide (700–800 ppm) and oxygen (FiO_2_ = 16%) were controlled and held constant to increase the reliability of the investigations. After the exercise test, venous blood was collected again to determine the post-exercise levels of 2,3-DPG and HIF-1α.

### 2.3. Incremental Exercise Test

The incremental test started with a load of 80 W, which was increased by 40 W every 3 min to exhaustion or until the participant was unable to maintain the minimal cadence of 60 rpm. At rest (3 min before the test) and during the exercise, heart rate (HR), minute ventilation (VE), breathing frequency (BF), oxygen uptake (VO_2_), expired carbon dioxide (VCO_2_) and respiratory exchange ratio (RER) were measured continuously with a fast gas analyzer (MetaLyzer 3B, Cortex, Leipzig, Germany) using the breath-by-breath method. The criteria for reaching VO_2max_ included a plateau in the level of VO_2_ or a gradual decrease in VO_2_ peak during the maximal workload, respiratory exchange ratio (RER) above 1.1 and blood lactate concentration (LA) above 8.0 mmol/l [41]. In the case of a last uncompleted stage, the maximal workload (WR_max_) was calculated from the following formula: WR_max_ = WR_k_ + (t/T × WR_p_) [42], where WR_k_ = previous workload, t = exercise duration with the work-load until premature failure, T = duration of each workload and WR_p_ = the amount of workload by which exercise intensity increased during the test. Cardiac output (Q) was estimated by VO_2max_ and was calculated by the following formula: Q = 100 × VO_2_/(5.72 + 10.5 × VO_2_/VO_2max_) using MetaSoft Studio (Cortex, Germany). Stroke volume (SV) was calculated as follows: SV= Q/HR.

At the end of each workload (the last 15 s), capillary blood samples were drawn from the fingertips to determine the blood LA levels (SUPER GL2, Dr. Müller Gerätebau GmbH, Freital, Germany). These data were used to evaluate the individual lactate threshold (LT) based on the D_max_ method [43]. Our previous studies [44,45] demonstrated that LT determined using the D-max method corresponds to the maximal lactate steady state (MLSS). Additionally, before and after the exercise test, fingertip capillary blood samples were drawn to analyze the acid-base balance and selected blood gas variables: oxygen saturation (SaO_2_), partial pressure of oxygen (pO_2_) and partial pressure of carbon dioxide (pCO_2_) in the blood and the oxygen partial pressure when hemoglobin is 50% saturated with oxygen (p50) as an indicator of hemoglobin oxygen affinity (OPTI CCA-TS2 Blood Gas and Electrolyte Analyzer, OPTI Medical Systems, Roswell, GA, USA). The same test protocol was applied to all the research series.

### 2.4. Determination of 2,3-DPG and HIF-1α

To prepare the samples for the 2,3-DPG measurement, a total of 2 mL of venous blood was collected in heparinized tubes, placed immediately on ice, deproteinized with 0.6 M perchloric acid to lyse erythrocytes, and neutralized with 2.5 M potassium carbonate. The supernatant was kept for at least 60 min in an ice bath and centrifuged at 3000× *g* for 10 min. The 2,3-DPG levels in the supernatant were measured using a commercial ELISA kit (No. MBS702898, My BioSource, San Diego, CA, USA) according to the manufacturer’s instructions. The 2,3-DPG assays were performed in two batches and in the range of 0.312–20 µmol/mL. The concentration of 2,3-DPG was calculated using a standard calibration curve obtained using 2,3-DPG standards, which allows the determination of the detection limit, the limit of quantification and the coefficient of variation. The minimum detectable dose of human 2,3-DPG is typically less than 0.078 μmol/mL. The intra-/interassay coefficients of variation were <8–10%. The 2,3-DPG levels were normalized to the corresponding hematocrit value from the same sample.

To prepare the samples for HIF-1α measurement, 2 mL of venous blood was collected and placed into tubes containing EDTA as an anti-coagulant. The blood was kept on ice and centrifuged to separate the plasma. The HIF-1α was measured using a commercial ELISA DuoSet kit (No. DYC1935-5, R&D Systems, Minneapolis, MN, USA) and Ancillary Reagent kit (No. DY007, R&D Systems, Minneapolis, MN, USA) following the manufacturer’s instructions. Standard calibration curves were obtained using HIF-1α protein standards, which allow the determination of the limit of detection, limit of quantification and coefficient of variation. HIF-1α assays were performed in two batches and in the range 125–8000 pg/mL. The minimum detectable dose of human HIF-1a is typically less than 0.078 μmol/mL. The intra-/interassay coefficients of variation were <5%. The post-exercise HIF-1α levels were corrected for the exercise-induced changes in plasma volume using the formula of Van Beaumont [46].

### 2.5. Supplementation with Sodium Phosphate

For 6 days during the first supplementation phase, the G1 group received tri-sodium phosphate (SP) at a dose of 50 mg/kg FFM per day, while the G2 group received a placebo in the form of 4 g of cellulose per day. The dose was divided into equal portions and administered four times a day at similar intervals. The subjects were blinded to the active and placebo allocation. The first phase of supplementation was followed by a 21 day washout period, during which the participants were not supplemented. Subsequently, the second supplementation phase began, in which SP was used in the G2 group and placebo in the G1 group. Throughout the experiment, all the participants received nutritional, training and supplementation recommendations.

The SP dose (50 mg/kg FFM per day) and supplementation time (6 days) were selected based on the methodology used in previous studies on ergogenic effects of phosphate salt supplementation in normoxia [24,25,27,28]. It was demonstrated that the dose of 3–5 g of SP per day applied over a period of 3–6 days is adequate to achieve sufficient serum phosphate levels and to ensure the expected supplementation benefits [19]. Doses greater than 6 g are usually avoided during supplementation, as they are associated with a reduction in the serum phosphate concentration by regulating its level through PTH. Doses below 3 g are generally considered too low to significantly increase serum phosphate levels [19,47,48]. The duration of the washout period (21 days) was based on the conclusions published by Cade et al. [20], who analyzed the changes in the 2,3-DPG levels observed following phosphate salt supplementation and suggested that the washout time should be 2 to 3 weeks to remove any carry-over effects from previous phosphate loading.

### 2.6. Statistical Analysis

The results of the study were analyzed using StatSoft Statistica 13.0 software (TIBCO Software Inc., Palo Alto, CA, USA). The statistical significance level for all the analyses was set at *p* < 0.05. The results were presented as mean ± SEM. Prior to all the statistical analyses, normality of the distribution of variables was checked using the Shapiro–Wilk test. The analysis of variance (ANOVA) for repeated measures (intervention (SP, placebo) × time [S1, S2]) was used to determine the differences in each of the dependent variables. When significant differences were found, the post hoc Tukey test was used. The relationships between the variables were analyzed using Pearson’s correlation coefficient. The effect sizes (ESs) were calculated from standardized differences (Cohen’s d units). The threshold values for Cohen ES statistics were considered to be small (0.20–0.60), moderate (0.60–1.20), large (1.20–2.0), very large (2.0–4.0), or extremely large (>4.0) [49]. The inter-individual variability in WR_LT_ and WR_max_ changes was expressed by the coefficient of variation (CV) using the formula CV = (SD/mean) × 100.

Based on an improvement in the LT workload (WR_LT_) and maximal workload (WR_max_) during the incremental exercise test following SP supplementation, all the subjects were retrospectively grouped as Responders or Non-Responders. The smallest worthwhile change was assumed to be an increase in WR_LT_ or WR_max_ of more than 3.0%, based on a previous study into the reliability of power in tests of exercise performance [50] and the smallest worthwhile change in performance in athletes [51,52].

## 3. Results

### 3.1. Exercise Performance and Cardiorespiratory Variables

The statistical analysis showed a significant interaction (intervention x time) for workload at lactate threshold (WR_LT_; F = 10.67, *p* < 0.01). The WR_LT_ increased due to SP supplementation by 2.8% (*p* < 0.01, d = 0.24). Similar changes were not observed after placebo ingestion. There were no significant changes in WR_max_, VO_2max_, HR_max_, SV_max_, Q_max_, VE_max_ or VO_2_/HR_max_ following SP supplementation (Table 1). The changes in VO_2max_ (∆VO_2max_) after phosphate loading significantly correlated (*p* < 0.001, r = 0.82) with the changes in oxygen pulse (∆VO_2_/HR_max_). The coefficient of variation for the changes in WR_LT_ and WR_max_ due to SP ingestion were 150% and 161%, respectively.

### 3.2. Acid-Base Balance and Blood Gas Analysis

There were no significant differences in acid-base balance response to exercise following the SP supplementation (Table 2). Exercise under hypoxic conditions caused a significant increase in p50 levels (*p* < 0.001); however, SP administration did not affect the magnitude of p50 changes (∆p50) due to exercise (Table 2). There were no statistically significant changes in resting p50 levels following SP supplementation (S1 vs. S2: SP 25.55 ± 0.21 vs. 25.40 ± 0.22 mmHg; Pl 25.42 ± 0.21 vs. 25.09 ± 0.22 mmHg).

### 3.3. 2,3-Diphosphoglycerate (2,3-DPG) and Hypoxia-Inducible Factor 1 alpha (HIF-1α)

There were no statistically significant changes in resting 2,3-DPG and HIF-1α levels following SP supplementation (Table 3). Exercise under hypoxic conditions caused a significant increase in the levels of 2,3-DPG (*p* < 0.001) and HIF-1α (*p* < 0.001); however, phosphate loading did not affect the exercise-induced changes in 2,3-DPG and HIF-1α.

The level of 2,3-DPG and the magnitude of 2,3-DPG changes (∆ 2,3-DPG) due to SP administration did not correlate with the changes in aerobic capacity or exercise performance (∆VO_2max_, ∆WR_max_, ∆WR_LT_). Additionally, 2,3-DPG levels and ∆ 2,3-DPG did not correlate with Pi levels or ∆ Pi.

### 3.4. Calcium–Phosphate Balance

The statistical analysis revealed a significant interaction (intervention x time) for serum Pi level at rest (F = 4.91, *p* < 0.05). The Pi level increased by 9.0% (*p* < 0.05, d = 0.78) following the SP supplementation. No significant changes were observed in resting serum Ca level and circulating PTH activity (Table 3). The level of resting Pi level and the magnitude of Pi changes due to SP ingestion did not correlate with the changes in exercise performance, cardiorespiratory variables or acid-base balance.

### 3.5. Responders vs. Non-Responders

The statistical analysis showed a significant interaction (group x time) for WR_max_ (F = 22.3, *p* < 0.001), WR_LT_ (F = 17.5, *p* < 0.001), VO_2max_ (F = 11.62, *p* < 0.001) and VO_2_/HR_max_ (F = 6.61, *p* < 0.05). In the Responders, an increase in WR_max_ by 2.8% (*p* < 0.001, d = 0.36), WR_LT_ by 5.7% (*p* < 0.001, d = 0.59), VO_2max_ by 4.1% (*p* < 0.01, d = 0.61) and VO_2_/HR_max_ by 3.7% (*p* < 0.01, d = 0.61) were registered after SP supplementation (Table 4). Similar changes were not observed in the Non-Responders. Additionally, the Responders demonstrated a significantly higher baseline and post-supplementation values of HR_max_ (by 6.6%, *p* < 0.05, d = 1.27 and by 7.7%, *p* < 0.01, d = 1.57, respectively), and lower baseline values of VO_2_/HR_max_ by 9.2% (*p* < 0.05, d = 1.22) than the Non-Responders. The age, body mass, and body compositions of participants did not differ between groups.

**Figure 2:**
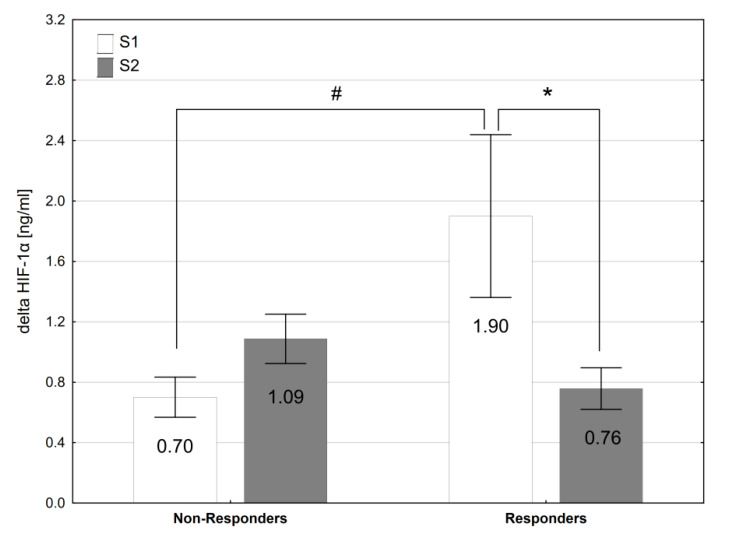
The exercise-induced changes in plasma HIF1α levels before (S1) and after (S2) sodium phosphate supplementation in Responders and Non-Responders. * *p* < 0.05 S1 vs. S2; ^#^ *p* < 0.05 Responders vs. Non-Responders.

The Responders did not differ from the Non-Responders in terms of baseline and post-supplementation resting levels of Pi, Ca, PTH, 2,3-DPG and HIF-1α (Table 5), as well as the values of hematological variables (Table 6). The exercise-induced changes in acid-base balance and blood gas were similar in both groups of participants. The analysis revealed a significant interaction (group × time) for exercise-induced changes in HIF-1α plasma level (∆HIF-1α; F = 8.50, *p* < 0.01). In the Responders, SP supplementation led to a significant reduction in HIF-1α response to exercise by 60% (*p* < 0.05, d = 0.92), which was not observed in the Non-Responders (Figure 2). Additionally, the Responders were characterized by a larger exercise-induced increase in HIF-1α plasma levels before SP supplementation (*p* < 0.05, d = 0.97) than the Non-Responders.

## 4. Discussion

Sodium phosphate supplementation has been proposed as an ergogenic aid for athletes exercising in normoxic conditions [19]. Accordingly, we expected that short-term phosphate loading would also be beneficial for aerobic performance in acute hypoxia. Our study revealed an increase by 2.8% in LT workload after 6 days of SP supplementation. The other indicators of aerobic performance, i.e., WR_max_, VO_2max_, HR_max_, SV_max_, Q_max_, VO_2_/HR_max_ and VE_max_, remained unchanged. These results showed that SP supplementation promotes improvements in hypoxic exercise performance only to a small extent and in relation to submaximal rather than maximal aerobic capacity. Our data indicated high inter-individual variability in response to SP supplementation. Therefore, the participants were retrospectively grouped as Responders and Non-Responders and, subsequently, the differences in physiological and biochemical response to phosphate loading between them were analyzed. The results showed that in the Responders, phosphate loading caused an increase in aerobic performance (VO_2max_, VO_2_/HR_max_, WR_max_, WR_LT_) and led to a reduction in the HIF-1α response to exercise. However, the mechanisms responsible for the observed changes seem to be other than those previously attributed to phosphate loading.

Several mechanisms have been proposed to explain the potential ergogenic effects of phosphate salt supplementation. Firstly, it has been proposed that phosphate loading enhanced 2,3-DPG concentration in the RBC. The increase in 2,3-DPG levels is a factor contributing to the shift of the oxygen dissociation curve (ODC) to the right and the reduction of Hb-O_2_ affinity [53], which promotes the O_2_ unloading to the peripheral tissues. With an increase in 2,3-DPG level after phosphate loading, an increase in VO_2max_ under normoxic conditions in athletes was observed [20,24].

The increase of 2,3-DPG levels is one of the first responses to hypoxic exposure [32,33,34] and is a consequence of an increase in blood pH and stimulation of RBC glycolysis [54]. Jain et al. [36] observed that during stays at an altitude of 3500 m, short-term (4 days) supplementation with sodium phosphate led to a greater increase in the level of 2,3-DPG in the blood (by 18%) than exposure to hypoxia itself. Furthermore, the phosphate loading supported an elevation of 2,3-DPG levels before ascent to altitude and maintenance of high 2,3-DPG levels during the early period of altitude adaptation [35,37]. However, it is not known whether the increase in 2,3-DPG levels resulted in an improvement in aerobic capacity and exercise performance in subjects under hypoxic conditions. 

Contrary to our hypothesis, we observed that 6 days of SP supplementation did not change the 2,3-DPG levels and Hb-O_2_ affinity, and did not improve VO_2max_ under acute hypoxic exercise in the overall group of participants. It should be noted that in Responders, the VO_2max_ increased by 4.1% after phosphate loading. Considering that acute hypoxic exposure to 2000–2500 m causes a reduction in VO_2max_ by ~10%, and half of the baseline level of VO_2max_ is recovered only after ~2 weeks of acclimatization to altitude [16], an improvement in aerobic capacity by 4% following phosphate loading in individual athletes can be decisive for success during competitions at altitude, especially when the time spent on altitude adaptation is shortened, e.g., for logistical reasons. However, a different mechanism was responsible for this improvement than that associated with the increase in 2,3-DPG level. The Responders did not differ from the Non-Responders in 2,3-DPG concentration at rest and after exercise (at baseline and after SP intervention), or in the range of 2,3-DPG changes following SP supplementation. Furthermore, in our study, the changes in the concentration of 2,3-DPG and VO_2max_ in hypoxia were not correlated.

The 2,3-DPG concentration is dependent on the Pi level in the blood [55]. Increased phosphate ingestion enhances the plasma Pi level and may increase the levels of intracellular Pi, resulting in the stimulation of glycolysis in RBC and higher 2,3-DPG levels [47]. A significant correlation between resting serum Pi concentration and 2,3-DPG levels after SP supplementation was previously described by Czuba et al. [24]. In our study, serum Pi levels increased after SP supplementation (by 9%), but this was insufficient to significantly increase 2,3-DPG and VO_2max_ improvement. We also found no correlation between serum Pi concentration and 2,3-DPG levels. Additionally, there was no relationship between the changes in Pi level and VO_2max_. These results are consistent with several previous reports in which, despite a significant increase in blood Pi levels, no changes in 2,3-DPG [26,56] or in VO_2max_ were observed [26,57]. It is considered that the Pi concentrations necessary to stimulate the glycolytic pathway and increase 2,3-DPG level are unphysiologically high [58]. Farber et al. [59] demonstrated that even with a large elevation in plasma Pi (>7 mg/dL) after phosphate intravenous infusion only a small increase Pi in RBC was observed. The short-term oral administration of phosphate leads to the elevation of plasma Pi levels only in the small range of 3.5–4.5 mg/dL [20,24,26,56]. Thus, it is suggested that an increase in plasma Pi after oral phosphate supplementation is ineffective at increasing 2,3-DPG levels and exerts a negligible physiological impact [26,47], which is also confirmed by the results of our study. 

Another mechanism proposed to explain the ergogenic effects of phosphate supplementation is enhancing the capacity of the buffering system in the intracellular fluids [19]. It has been suggested that SP supplementation may increase hydrogen phosphate (HPO_4_^−^) concentrations, which could improve the ability to buffer hydrogen ions (H^+^), slow down the pH drop and, consequently, increase work capacity during intense exercise [60]. During exercise under hypoxic conditions, the reduction in oxygen availability causes earlier reliance of ATP production on anaerobic glycolysis, leading to larger accumulation of H^+^ [61,62]. Therefore, exercise-induced acidosis in hypoxia is more severe than in normoxia [4,63]. Furthermore, the ability to remove H^+^ can diminish during the first 24–48 h of altitude acclimatization, due to lower intracellular and extracellular bicarbonate concentration, as a result of respiratory alkalosis and renal excretion of bicarbonate [64,65,66]. Theoretically, the improvement in the buffering capacity of muscle cells by phosphate loading may be helpful during intense exercise in hypoxia. Some studies conducted in normoxia revealed an increase in anaerobic threshold after phosphate loading [21,23,24,56]. However, improvement in acid-base balance during normoxic exercise has not been confirmed [24,26]. Our study was the first to investigate this issue in relation to exercise in hypoxia. We found that the exercise-induced changes in acid-base balance remained unchanged following SP supplementation. Given that the participants did not achieve a higher WR_max_, it can be concluded that phosphate loading did not improve buffering capacity during exercise to exhaustion in hypoxia. On the other hand, we noted a slight shift of LT to higher workloads. Together, these results suggest that phosphate loading may be helpful in moderate- but not in high-intensity exercise when the H^+^ production rate and intracellular acidosis are high. However, it should also be noted that the improvement in LT observed in our study was <3% (average increase by ~6 W), which is within the range for day-to-day biological variation and methodological error of anaerobic threshold [67,68]. Additionally, the effect size for changes of WR_LT_ is small, so the practical significance of this result may be negligible. Therefore, despite the statistical significance, this result should be interpreted with caution.

The ergogenic benefits associated with phosphate salt supplementation may also be attributable to improved myocardial efficiency, which is explained by enhanced myocardial contractility as a result of an increase in the levels of cardiac cell ATP [19,56]. When myocardial contractility improves, the heart can eject more blood out into the vascular system, thus increasing the SV, resulting in greater and more efficient oxygenation of muscles during exercise. It is recognized that increasing SV is much more efficient than increasing HR during exercise in terms of myocardial oxygen demand [69], which may be crucial in hypoxic conditions, when oxygen availability is limited. Our recent research demonstrated that phosphate loading leads to a decrease in HR, an increase in SV and an improvement in oxygen pulse (VO_2_/HR) during exercise at low-to-moderate intensity (≤LT) under moderate hypoxia [38]. Similar benefits after phosphate loading were previously reported in normoxia. For example, it was found that SP supplementation led to a reduction in resting and maximal HR and to improvement in VO_2_/HR_max_ in elite mountain bike cyclists [23,24]. Furthermore, increases in end-diastolic volume, SV and cardiac output during endurance exercise in normoxia were also reported [56]. These adaptations were accompanied by improved aerobic capacity and exercise performance in athletes [23,24,56]. In the present study, HR_max_, VO_2_/HR_max_, SV_max_ and Q_max_ did not change after phosphate loading in the overall group of participants. These results indicated that SP supplementation is not beneficial for improving myocardial efficiency at maximal aerobic exercise intensity (at 100% VO_2max_) in hypoxia.

It is interesting that in the Responders, SP supplementation led to an improvement in VO_2_/HR_max_. The oxygen pulse (VO_2_/HR) is the volume of oxygen ejected with each heart ventricular contraction. According to the Fick equation, oxygen pulse is the product of SV and arterial to mixed venous oxygen difference, and it provides an estimation of SV and peripheral vascular perfusion/extraction. The increase in oxygen pulse reflects central and peripheral adaptation to exercise [70]. Because, in our study, SV_max_ did not change after phosphate loading, we speculate that the improvement in aerobic capacity that occurred in the Responders may be associated with peripheral mechanisms affecting VO_2max_, for example, with enhanced peripheral oxygen extraction capacity [71], but induced by factors other than better Hb-O_2_ offloading. Currently, the mechanisms responsible for these adaptations and the role of phosphates in them remain unknown.

We found that in the Responders, the exercise-induced increase in HIF-1α was reduced after SP supplementation. HIF-1α is a well-established hallmark of hypoxia signaling and it plays an important role in both the acute responses and the long-term cellular adaptations to oxygen deficiency [72,73]. It has been demonstrated that hypoxia and exercise stimulate HIF-1α activation in response to a reduction in intracellular oxygen tension in skeletal muscles [74,75,76]. Thus, our results suggest that the Responders’ muscle tissue oxygenation during exercise in hypoxia was improved by SP supplementation. Lindholm and Rundqvist [77] proposed that the downregulation of HIF-1α activity in skeletal muscle enables enhanced oxidative metabolism and may be beneficial for endurance performance. In our study, we did not determine the effect of phosphate loading on oxidative enzyme activity and mitochondrial function; thus, future research should aim to verify and explain these potential benefits.

## 5. Practical Applications and Future Research

We found that short-term SP supplementation may provide ergogenic benefits to certain individual endurance athletes. Since SP supplementation Responders had a higher baseline HR_max_ and a lower VO_2_/HR_max_, as well as a higher baseline HIF-1α response to exercise, it can be expected that greater benefits from phosphate loading may be obtained by athletes with lower central (cardiovascular) and/or peripheral adaptation induced by endurance training. These findings may be important not only for competitive athletes but also for mountain tourists and active people who practice sports recreationally in mountain areas. It is possible that untrained people will be more responsive to the ergogenic effects of phosphate salt supplementation in a hypoxic environment when compared with trained athletes. This seems to be an interesting question for future investigations, because an improvement of aerobic capacity in hypoxic conditions in a short time by the use of phosphate loading would serve to improve health safety in people going to mountains, at moderate altitudes (<3000 m), especially since such trips often take place without prior altitude adaptation.

Importantly, our study, like previous reports [24,26], shows that short-term SP supplementation does not cause hypocalcemia, and does not change the PTH level, which indicates that the proposed phosphate loading protocol does not disturb the phosphate-calcium balance. Additionally, the applied dose of 50 mg/kg FFM of tri-sodium phosphate per day appears to be generally safe in subjects without kidney disease. Only one participant in our experiment reported side effects of SP supplementation, in the form of stomach pain, after three days of phosphate loading. These findings indicate that the effectiveness of short-term phosphate loading in individual athletes may be tested in sports practice without the undue risk of negative health effects.

In our study, we analyzed the effect of SP supplementation on aerobic capacity in athletes during a single exercise bout in acute hypoxia. It would also be interesting from the point of view of sports practice to investigate whether SP supplementation can be helpful during the first days of staying at altitude, in order to improve hypoxic adaptation, as suggested in early research on phosphate loading [35,36,37], and to achieve a faster retrieval of aerobic exercise capacity at altitude. It is also worth analyzing whether SP may be beneficial in the prevention/attenuation of altitude-related diseases due to the mechanism associated with the alteration of the HIF-1α response to hypoxia. Clarification of these issues will be helpful in the acclimatization phase before an altitude competition, during the early stages of high-altitude expeditions or during altitude training.

## 6. Study Limitations

The present study features certain limitations. Firstly, during the experiment, the participants were not accommodated in the same place and did not follow the same training program. However, the athletes received nutritional, training and supplementation recommendations that were constantly monitored. The participants were also instructed to follow the same diet and to abstain from caffeine, alcohol, and strenuous exercise for 24 h before all the trials. Secondly, we used an incremental exercise test to assess aerobic capacity in cyclists, but carrying out a time trial simulation additionally would better reflect the effect of SP supplementation on exercise performance and the practical significance of physiological changes registered under the influence of phosphate loading. Thirdly, when designing the study, we focused mainly on the adaptive mechanism related to the increase in 2,3-DPG levels and did not take into account more accurate and advanced methods of measuring myocardial efficiency. Thus, it would be necessary to include such an analysis in future studies. As we suggested above, further research should also consider analyzing the effects of phosphate salts on oxidative enzyme activity, mitochondrial function and muscle tissue oxygenation during exercise both in normoxia and hypoxia. 

## 7. Conclusions

This was the first study to investigate the effect of phosphate loading on aerobic capacity under acute hypoxic conditions. We found that phosphate loading did not trigger any of the previously proposed adaptive mechanisms, such as increasing 2,3-DPG levels, improving buffering capacity, or enhancing myocardial efficiency and did not contribute to the improvement of aerobic capacity under moderate hypoxia in competitive athletes. However, our results also showed that the response to phosphate salt ingestion varies among individuals. In certain individuals, an improvement in maximal oxygen uptake and power output at submaximal (at LT) and maximal (at VO_2max_) exercise intensity in the range of 3–5% can be expected following phosphate loading. This benefit applies primarily to athletes with less training-induced central and/or peripheral adaptation. 

## Figures and Tables

**Table 1:** Exercise performance and cardiorespiratory variables before (S1) and after (S2) sodium phosphate and placebo supplementation.

Variables	Sodium Phosphate		Placebo	
	Before (S1)	After (S2)	Before (S1)	After (S2)
WR_max_ (W)	310.7 ± 6.2	314.7 ± 6.3	303.5 ± 6.2	307.5 ± 6.3
WR_LT_ (W)	227.3 ± 5.4	233.6 ** ± 5.7	228.2 ± 5.4	227.3 ± 5.7
VO_2max_ (L·min^−1^)	3.49 ± 0.06	3.54 ± 0.06	3.53 ± 0.06	3.49 ± 0.06
HR_max_ (bpm)	178 ± 2.3	177 ± 2.3	178 ± 2.3	178 ± 2.3
SV_max_ (ml)	108.4 ± 2.2	108.7 ± 2.3	108.3 ± 2.2	110.1 ± 2.4
Q_max_ (L·min^−1^)	19.3 ± 0.3	19.2 ± 0.3	19.5 ± 0.3	19.5 ± 0.3
VE_max_ (L·min^−1^)	170.5 ± 3.1	169.6 ± 3.0	168.5 ± 3.1	171.5 ± 3.0
VO_2_/HR_max_ (ml·bpm^−1^)	19.6 ± 0.4	20.0 ± 0.4	19.9 ± 0.4	19.6 ± 0.4

WR_max_—maximal workload, WR_LT_—workload at lactate threshold, VO_2max_—maximal oxygen uptake, HR_max_—maximal heart rate, SV_max_—maximal stroke volume, Q_max_—maximal cardiac output, VE_max_—maximal minute ventilation, VO_2_/HR_max_—maximal oxygen pulse; ** *p* < 0.01 S1 vs. S2.

**Table 2:** Exercise changes in acid-base balance and blood gas before (S1) and after (S2) sodium phosphate and placebo supplementation.

Variables	Sodium Phosphate		Placebo	
	Before (S1)	After (S2)	Before (S1)	After (S2)
∆ LA (mmol·L^−1^)	11.78 ± 0.55	12.76 ± 0.50	12.09 ± 0.55	12.50 ± 0.50
∆ pH	−0.187 ± 0.013	−0.208 ± 0.013	−0.174 ± 0.013	−0.197 ± 0.013
∆ BE (mmol·L^−1^)	−14.05 ± 0.61	−14.65 ± 0.64	−13.35 ± 0.61	−14.30 ± 0.64
∆ HCO_3_ (mmol·L^−1^)	−12.49 ± 0.45	−12.62 ± 0.46	−11.96 ± 0.45	−12.49 ± 0.46
∆ sO_2_ (%)	−5.13 ± 0.99	−5.72 ± 0.74	−5.12 ± 0.99	−5.48 ± 0.74
∆ pO_2_ (mmHg)	−0.08 ± 1.57	−0.10 ± 1.25	−0.10 ± 1.53	−0.55 ± 1.22
∆ pCO_2_ (mmHg)	−8.15 ± 0.62	−7.02 ± 0.54	−8.25 ± 0.62	−7.75 ± 0.54
∆ p50 (mmHg)	5.03 ± 0.45	5.42 ± 0.45	4.49 ± 0.45	5.22 ± 0.45

LA—lactate concentration, BE—base excess, HCO_3_—bicarbonate, sO_2_—blood oxygen saturation, pO_2_—partial pressure of oxygen, pCO_2_—partial pressure of carbon dioxide, p50—the oxygen partial pressure when hemoglobin is 50% saturated with oxygen.

**Table 3:** Biochemical variables before (S1) and after (S2) sodium phosphate and placebo supplementation.

Variables	Sodium Phosphate		Placebo	
	Before (S1)	After (S2)	Before (S1)	After (S2)
Pi rest (mg·dL^−1^)	3.10 ± 0.07	3.38 * ± 0.08	3.28 ± 0.07	3.30 ± 0.08
Ca rest (mg·dL^−1^)	9.54 ± 0.06	9.51 ± 0.06	9.52 ± 0.06	9.46 ± 0.06
PTH rest (pg·mL^−1^)	30.36 ± 1.97	31.25 ± 2.14	30.32 ± 1.97	30.49 ± 2.14
2,3-DPG rest (mmol·L er.^−1^)	6.19 ± 0.51	7.29 ± 0.36	7.03 ± 0.54	6.45 ± 0.38
2,3-DPG max (mmol·L er.^−1^)	8.61 ^#^ ± 1.17	9.80 ^#^ ± 0.86	11.86 ^###^ ± 1.29	10.03 ^###^ ± 0.85
HIF-1α rest (ng·mL^−1^)	1.66 ± 0.39	1.90 ± 0.42	2.12 ± 0.40	2.30 ± 0.43
HIF-1α max (ng·mL^−1^)	2.96 ^###^ ± 0.57	2.82 ^###^ ± 0.51	3.38 ^###^ ± 0.59	3.87 ^###^ ± 0.49

Pi—inorganic phosphate, Ca—serum calcium, PTH—parathyroid hormone, 2,3-DPG—2,3-diphosphoglycerate, HIF-1α—hypoxia-inducible factor 1 alpha; rest—before exercise, max—immediately after exercise; * *p* < 0.05 S1 vs. S2; ^#^ *p* < 0.05; ^###^ *p* < 0.001 rest vs. max.

**Table 4:** Exercise performance and cardiorespiratory variables in Responders and Non-Responders before (S1) and after (S2) sodium phosphate supplementation.

Variables	Responders (*n* = 11)		Non-Responders (*n* = 11)	
	Before (S1)	After (S2)	Before (S1)	After (S2)
WR_max_ (W)	307.6 ± 9.2	316.1 *** ± 9.2	313.8 ± 9.2	313.3 ± 9.2
WR_LT_ (W)	223.6 ± 8.4	236.4 *** ± 9.0	230.9 ± 8.4	230.9 ± 9.0
VO_2max_ (L·min^−1^)	3.43 ± 0.09	3.57 ** ± 0.08	3.54 ± 0.09	3.51 ± 0.08
HR_max_ (bpm)	184 ^#^ ± 2.7	184 ^##^ ± 2.5	172 ± 2.7	171 ± 2.5
SV_max_ (mL)	103.7 ± 2.9	103.6 ± 3.2	112.4 ± 2.7	112.9 ± 2.9
Q_max_ (L·min^−1^)	19.3 ± 0.4	19.2 ± 0.4	19.2 ± 0.5	19.2 ± 0.5
VE_max_ (L·min^−1^)	170.4 ± 4.6	170.0 ± 4.3	170.6 ± 4.6	169.2 ± 4.3
VO_2_/HR_max_ (ml·bpm^−1^)	18.7 ^#^ ± 0.5	19.4 ** ± 0.5	20.6 ± 0.5	20.6 ± 0.5

WR_max_—maximal workload, WR_LT_—workload at lactate threshold, VO_2max_—maximal oxygen uptake, HR_max_—maximal heart rate, SV_max_—maximal stroke volume, Q_max_—maximal cardiac output, VE_max_—maximal minute ventilation, VO_2_/HR_max_—maximal oxygen pulse; ** *p* < 0.01, *** *p* < 0.001 S1 vs. S2; ^#^ *p* < 0.05, ^##^ *p* < 0.01 Responders vs. Non-Responders at the same measuring point (S1 or S2).

**Table 5:** Biochemical variables at rest in Responders and Non-Responders before (S1) and after (S2) sodium phosphate supplementation.

Variables	Responders		Non-Responders	
	Before (S1)	After (S2)	Before (S1)	After (S2)
Pi (mg·dL^−1^)	3.07 ± 0.10	3.44 ± 0.11	3.14 ± 0.10	3.31 ± 0.11
Ca (mg·dL^−1^)	9.58 ± 0.08	9.56 ± 0.09	9.50 ± 0.08	9.47 ± 0.09
PTH (pg·mL^−1^)	27.67 ± 2.69	30.58 ± 3.09	32.80 ± 2.57	31.85 ± 2.95
2,3-DPG (mmol·L er.^−1^)	6.54 ± 0.57	7.39 ± 0.58	5.83 ± 0.57	7.19 ± 0.58
HIF-1α (ng·mL^−1^)	1.57 ± 0.52	1.83 ± 0.66	1.76 ± 0.52	1.96 ± 0.66

Pi—inorganic phosphate, Ca—serum calcium, PTH—parathyroid hormone, 2,3-DPG—2,3-diphosphoglycerate, HIF-1α—hypoxia-inducible factor 1 alpha.

**Table 6:** Hematological variables at rest in Responders and Non-Responders before (S1) and after (S2) sodium phosphate supplementation.

Variables	Responders		Non-Responders	
	Before (S1)	After (S2)	Before (S1)	After (S2)
RBC (T·L^−1^)	5.02 ± 0.08	5.01 ± 0.09	4.88 ± 0.07	4.80 ± 0.08
Ret (‰)	12.7 ± 0.96	12.8 ± 0.65	12.6 ± 0.86	12.0 ± 0.59
[Hb] (g·dL^−1^)	14.86 ± 0.21	14.97 ± 0.27	14.68 ± 0.19	14.45 ± 0.24
Hct (%)	43.6 ± 0.54	43.8 ± 0.69	42.8 ± 0.49	42.3 ± 0.62

RBC—red blood cell count, Ret—reticulocytes, [Hb]—hemoglobin concentration, Hct—hematocrit value.

## Data Availability

The data presented in this study are available on request from the corresponding author.
